# The introduction of group antenatal care in northern Nigeria: an implementation research study analysing changes in service use

**DOI:** 10.1136/bmjgh-2025-022038

**Published:** 2026-07-14

**Authors:** Abudulhamid Abubakar, Grace Yisa, Adeola Seweje, Joseph Odu, Costa Atori, Uche Ralph Opara, Rondi Anderson

**Affiliations:** 1 Project HOPE, Abuja, Nigeria; 2 Project HOPE, Timmins, Ontario, Canada; 3 Project HOPE, Rockville, Maryland, USA; 4 Project HOPE, Washington DC, Virginia, USA

**Keywords:** Africa, Maternal health, Public Health

## Abstract

**Introduction:**

With one of the highest rates of maternal mortality in the world, improving antenatal care for the poorest communities is a high priority in Nigeria. In 2022, Project HOPE partnered with the Nigerian Ministry of Health to implement group antenatal care, a WHO-recommended health system strengthening intervention for improving pregnancy outcomes in Niger State.

**Methods:**

The project trained 300 health workers and established group antenatal care in 150 health facilities. Training and initiation was staggered over September, October and November of 2022. Over 17 000 women participated in 1054 antenatal care groups from September 2022 to March 2023. We carried out an implementation research study to examine service utilisation rates and explore contributing factors within the project. Using an interrupted time series analysis, we examined project effects on antenatal contacts, facility births, live births, malaria positivity, postpartum contraception and postnatal care.

**Results:**

The analysis found statistically significant increases in first and fourth antenatal visits, facility births and live births following the intervention.

**Conclusion:**

Despite significant challenges, including a shortened implementation period, the project increased service use. We assert that health system approaches were key influencing factors, such as the use of local master trainers, broad stakeholder engagement, strong monitoring and evaluation and the inclusion of the group antenatal care model in national guidelines. Noted challenges included inadequate skilled birth attendants and lack of antenatal care commodities, though stop gap measures addressed certain supply issues such as a lack of blood pressure cuffs.

**WHAT IS ALREADY KNOWN ON THIS TOPIC**

**WHAT THIS STUDY ADDS**

- The results show that group antenatal care that is part of a broad partnership model and includes facility mentorship and adequate supplies for care delivery can increase evidence-based practices that lower maternal mortality.

**HOW THIS STUDY MIGHT AFFECT RESEARCH, PRACTICE OR POLICY**

- This study contributes to the established knowledge on group antenatal care as an evidence-based maternal health intervention. It examines the roles of stakeholder engagement, broad system and service delivery inputs and mentorship in supporting successful rollout in a fragile, insecure setting*.*

## Introduction

 Nigeria is responsible for 29% of global maternal mortality.^1^ With the third highest maternal mortality ratio of 993/100 000 and a population of over 232 million, maternal mortality is slow to decline.^2 3^ A major contributor to this is gaps in access to quality maternal healthcare, particularly for rural women. More than 15% of pregnant women in almost 2/3 of Nigeria’s states receive no antenatal care (ANC), higher than global averages.^4^ In 2016, WHO issued recommendations on ANC for a positive pregnancy experience.^5^ The recommendations prioritised ‘person-centred care’ as essential for improved health and well-being. Communication and support at ANC contacts are highlighted as key to improving quality and utilisation of healthcare services.

Group antenatal care (G-ANC), a WHO-recommended health system strengthening intervention, is a facility-based alternative model designed to improve women’s receipt of quality healthcare during and after pregnancy. It includes having a minimum of eight ANC contacts, giving birth in a health facility rather than at home, taking malaria prophylaxis, attending mother and baby postnatal visits and using contraception following childbirth.^5 6^ G-ANC is a service delivery model in which pregnant women with similar gestational ages receive clinical assessment, education and peer support together in small groups, in addition to one-to-one visits. Sessions usually last 1–2 hours. The model combines three components delivered in an integrated session: (1) individual visits, (2) facilitated group learning and (3) peer support and discussion. Each woman receives private clinical assessments, but much of the visit occurs in a shared group setting. Ideally, there is consistent membership across the sessions. The G-ANC model is meant to empower women by enhancing peer-to-peer support and self-care.

G-ANC strengthens health systems by addressing multiple system components. Group learning provides women with an opportunity to not only learn from providers but also to learn from their peers and from their peers’ interactions with providers.^7^ Organised group sessions are considered an effective means to build health literacy in a culturally competent fashion.^8^ Group sessions can also increase provider productivity and efficiency by enabling them to communicate the same information once in a group setting rather than individually with each woman. Some studies have found that women report increased satisfaction with ANC services when provided in a G-ANC format, contributing to better care continuity. Overall, G-ANC is associated with improved care quality, higher ANC care seeking and higher facility birth rates, which are in turn associated with decreased maternal mortality.^9^

This article is an implementation research study of the introduction of G-ANC in 150 of Niger State’s 274 primary healthcare (PHC) facilities. With close to 7 million people, the population of Niger State is widely dispersed across 4100 hard-to-reach settlements.^10^ Primary health facilities have a severe shortage of skilled human resources with associated gaps in functionality. Only 60% of women in Niger State receive any ANC care, despite the national adoption of the WHO’s minimum of eight ANC contacts.^4^ Only 2% of women in Niger have full ANC coverage. Prior to the introduction of G-ANC through this project, several successful G-ANC trials were carried out. The project thus aimed to implement large-scale G-ANC services in Niger State, and thereby improve care quality and health seeking. In this paper, we sought to understand the project’s effects on maternal health measures after 6 months of implementation. We used an interrupted time series analysis of routine facility data to measure project effects on antenatal contacts, facility births, live births, malaria positivity, postpartum contraception and postnatal care (PNC). Our interpretation of the results connects them with relevant aspects of implementation.

## Methods

### Study setting

#### Project design

As part of a national system strengthening initiative in May of 2022, Project HOPE with the Nigerian Ministry of Health commenced the G-ANC project in Niger State. Inception and co-creation meetings with key stakeholders were held. An initial desk review of successful G-ANC practices was completed, and based on that, the national and state health policy, annual operation plan and budget were revised to include a G-ANC model of care. To evaluate the project, monitoring and supervision systems were strengthened including a strong focus on quality data collection and reporting. For implementation of the project and accurate monitoring, key tools were developed, adapted and contextualised. The tools included an orientation package for local health authorities, a harmonised mentoring checklist, job aids for implementation of G-ANC and a scheduling tool for planning G-ANC as prior to this initiative ANC was only by drop-in. Data collection registers and summaries were used.

To maximise impact, collaborations were formed with partner and government initiatives that complemented the G-ANC system strengthening intervention. The collaborations were formed with the Malaria Consortium, the World Bank Accelerated Nutrition Programme, Plan International, state programmes for financing health facility equipment, state health insurance schemes, the Department of Health Planning Research and Statistics and community outreach workers. These complementary programmes distributed supplies for malaria prevention, ensured the sustainability of critical equipment for ANC, increased enrolment in health insurance and supported community outreach. The partnerships filled critical gaps at the service delivery level that were necessary for successful implementation, such as ensuring the availability of ANC supplies and logistics. This included bringing community-based services to facilities to ensure a larger reach, and training partners on quantification for the needed commodities. The State Health Education unit and media were also involved in key messaging for visibility and proper utilisation through airing of jingles in indigenous languages.

#### Baseline assessment

In preparation for this project, a mixed-method baseline facility readiness assessment was conducted. Interviews were held with eight policy makers followed by visits to 274 health facilities. Two hundred and sixty-four facility managers and healthcare providers, and 278 pregnant women (about one per facility) identified during data collection facility visits participated in computer-assisted personal interviews. The survey was programmed using the CommCare platform. Descriptive analysis was applied to the quantitative data, while thematic analysis was used to analyse the qualitative data.

Almost all the targeted facilities provided ANC, intrapartum care and PNC, although hours were limited with only 67% providing 24/7 care. Ninety per cent of health facilities had adequate space for ANC services and 50% had adequate seating, hand wash basins with soap and water and drinking water. Notably less than 10% had devices for measuring blood pressure. Only 6% of the health workers were nurses or midwives, with 94% being ancillary or community outreach staff. Seventy-five per cent of health workers had received short (less than 1 week) reproductive, maternal, newborn and child health in-service training at some point in their career.

Individual ANC was the predominant care model in the state. Forty-eight per cent of interviewed women had given birth to their most recent child at a PHC and 60% of pregnant women were planning a facility birth. Fifty-five per cent of pregnant women had at least one ANC visit with only 13% and 9% reaching the fourth and eighth visits. Seventy per cent of pregnant women received at least one malaria prophylaxis treatment and 9% received insecticide-treated bed nets. All facilities had data officers, and reporting was ongoing to various degrees. Additional findings included inadequate provider skills, inconsistent supplies of drugs and other consumables and fees for services affecting uptake including for facility birth. While the baseline assessment provided a picture of service provision and utilisation at the project’s outset, this study’s results are not directly comparable because the data were sourced from the national health information system that sampled all women attending the project facilities.

#### Implementation

In September of 2022, training of healthcare workers (HCWs) on the provision of G-ANC was initiated followed by the roll-out of the new G-ANC model. Initiation was staggered across facilities from September to November 2022, as each facility began immediately after HCW training. Enabling environments for care provision were strengthened through mentoring and by networking with national schemes for enrolling women in health insurance and supporting provision of malaria medicines. To evaluate the project, monitoring and supervision systems were strengthened.

In February 2023, implementation abruptly stopped because the donor decided to shift priorities. The funding stop was not related to project outcomes. Over the 6 months of implementation, 301 health workers were trained and G-ANC was initiated in 150 health facilities.

#### Training, support, mentoring and supervision

Several types of training at both state and local levels were initiated. They included training for policy makers, data collectors, community workers, healthcare providers, supportive supervisors, mentors and managers. In addition to training, the project provided technical support and facilitated skills transfer to local health authorities. Ongoing quality improvement systems, mentoring and supportive supervision were strengthened. Training included:

##### Data management

Project HOPE trained all 274 government designated monitoring and evaluation focal persons on DHIS2 and data management. Data management training was also conducted for 1051 HCWs and managers.

##### Community outreach

Four hundred and seven community outreach workers, promotors and supporters (30 per LGA) were trained on demand creation and referral to G-ANC implementing facilities. The training utilised a variety of modalities including presentations, role plays, group work and plenary sessions. In addition, advocacy visits and dialogues were conducted for community gatekeepers, including district heads and women and youth leaders.

##### G-ANC provision

Forty master trainers were certified after a 5 day training. In the first month following this, master trainers trained 112 HCWs, establishing G-ANC in 34 PHCs. In the second month, they trained 109 more HCWs, which established G-ANC in 57 more facilities.

In total, master trainers trained 301 HCWs, including 13 community health officers, 249 community health extension workers and 31 nurse-midwives, two per healthcare facility. This resulted in 151 primary care facilities providing G-ANC. Trainings covered the G-ANC methodology, including encouraging pregnant women to attend ANC visits, give birth in a facility and enrol in G-ANC. Following the trainings, master trainers visited the health facilities on ANC clinic days to provide hands-on support enrolling women into G-ANC and conducting the sessions.

The G-ANC model followed the global guidelines by recruiting small groups of women with similar gestational ages to meet together for the duration of the pregnancy. Components of care included group teaching and experience sharing, shared self-care such as weight and blood pressure measurement and individual meetings with care providers.

##### Mentors and supportive supervisors

To ensure enabling environments and support problem solving, 40 local government maternal and child health coordinators and monitoring officers were trained as mentors. An additional 15 PHC directors and five state-level supervisors were also trained on G-ANC supervision. To train the mentors and supervisors, a Project HOPE programme manager and senior technical officer led a 1 day meeting with key government stakeholders. Mentors and supervisors reviewed a visit checklist and capacity-strengthening indicators. All facilities were visited by mentors at least monthly and mentoring checklists were filled out at every visit to guide as a job aid and provide feedback to the programme.

### Supporting logistics and supplies

Facility officers in charge were given technical support on accessing the Basic healthcare Provision Fund under Nigeria’s National Health Act, which was designed to improve PHC. The scheme includes resources for procurement of essential lifesaving commodities. Through this initiative, it was hoped that all health facilities would have at least one blood pressure cuff, though the baseline assessment results demonstrated otherwise. Improving supply chain management for commodities and equipment was thus prioritised through training and technical support for proper quantification, procurement and distribution of commodities. In addition, some blood pressure cuffs were donated by the project to fill gaps until all government provided supplies could be distributed. This was necessary for effective delivery of ANC services. The project also copied over 8000 government data collection tools, teaching materials, and service tracking forms for pregnant women.

### Implementation challenges

Most challenges involved insecurity. In certain areas of Niger State kidnapping, banditry, cattle rustling and armed robbery were common. Some HCWs needed to travel to participate in trainings in safer areas, and some selected facilities could not participate because of the security concerns. The gaps in resources identified in the baseline assessment affected the project implementation. They included inadequate human resources, particularly midwives and nurses and availability of essential equipment and supplies. Health facilities also did not have scheduling systems, which presented challenges for planning G-ANC and individual ANC sessions. Initiatives to leverage state programmes for facility procurement of much needed commodities met barriers, and in the duration of the programme only saw small success. Women were not aware of the much-needed government health coverage, and adequate support for enrolling all communities was not present. Because of gaps in resources and inadequate coverage, many very poor women need to pay out of pocket for basic healthcare, perpetuating hesitance to seek lifesaving interventions. In addition, outreach workers were not compensated on schedule, causing demotivation and interfering with aspects of programme implementation.

### Study design and objectives

This study was part of a larger project approved by the Ministry of Health and Family Welfare and funded by the Bill and Melinda Gates Foundation under project ID BGD10MWC. Data analysis was supported under this project; however, writing the manuscript was carried out independently without funding. The primary objective was to conduct implementation research to understand the effects of the large-scale, short G-ANC intervention. The study examined routinely collected facility data with an interrupted time series analysis. The documentation of the project’s design and implementation informed the interpretation of the results.

### Patient involvement

Women who had participated in the G-ANC intervention and their families were not involved in the design and conduct of the study. However, nearly 300 women participated in interviews as part of the baseline assessment. As such, their knowledge and experiences directly informed the project design. Women and their families were also central to dissemination of the baseline information, which helped to motivate community involvement during and beyond the study. In addition, the low numbers of ANC contacts and the barriers to receiving maternity care in a health facility shared by women in the baseline informed the selection of all the measures assessed in the analysis.

### Data sources and collection

Data collection and analysis were conducted by the project monitoring and evaluation team. Two tools were developed for this purpose: a register for G-ANC clients and a monthly summary derived from that register. The register tracked a range of service delivery and health outcome indicators, including training, number of cohorts, community outreach activities, first and fourth ANC visits, total and live births, malaria positivity among febrile covid patients, postpartum contraceptive use and postnatal clinical visits for both mothers and newborns.

Quantitative data on the implementation of the programme and changes in service utilisation after the initiation were collected to understand the intervention’s effects. Data were collected from all 150 project facilities as part of programme reporting on all women who attended maternity services at the facilities providing G-ANC. This included women who did not attend G-ANC. Quantitative data on service utilisation were sourced from the DHIS2. Qualitative interviews and project documentation informed project design and interpretation of study results, but were not formally reported in this study.

DHIS2 data in Nigeria and in other sub-Saharan African countries has known limitations. Various studies document under-reporting, incomplete records, inconsistencies and the presence of data outliers, as well as efforts to improve data quality.^11^,^14^ While data quality limitations are acknowledged in this study, they were also recognised in the baseline assessment and significant continuous quality improvement capacity building efforts were instituted as part of project implementation. Ongoing mentoring and supportive supervision were carried out within the project for data quality improvement and data quality assessments were conducted at all project facilities.

Within the data set used for analysis, all facilities reported data for all months of project implementation. Potential extreme values were identified using the IQR rule within facilities. Across variables used in this study, 4.2%–5.9% of observations were flagged. Review of facility-level time series indicated that these values were plausible and may reflect expected variation in service volume rather than data entry errors; therefore, no observations were excluded. This degree of variability is typical in facility-level count data and was addressed through the use of negative binomial models, which account for overdispersion. Missing values were counted as zeros.

### Quantitative analysis

To evaluate the impact of the G-ANC intervention, an interrupted time series analysis using linear regression was conducted using R version 4.5.0.^15^ All pregnant women receiving ANC at the healthcare facilities between September 2022 and March 2023 were included in the evaluation although only around 50% had received G-ANC. Interrupted time series models were fitted to evaluate changes in maternal and newborn health indicators following the intervention. Eight outcomes were examined: first ANC visit, fourth ANC visit, facility births, live births, malaria test positivity (positive tests among persons with fever), postpartum contraceptive use, postnatal clinical visit (mother), and postnatal clinical visit (newborn).

The interrupted time series specification included the following three terms:

- Time (to assess the underlying trend).
- Level (to estimate the immediate effect of the intervention).
- Slope (to estimate the change in trend postintervention).

Data were reported monthly. The intervention was implemented at different times across facilities, resulting in a staggered rollout. To account for this, the interrupted time series model incorporated facility-specific intervention start dates, aligning the level and slope change indicators relative to each facilities’ intervention timeline. This approach enabled estimation of the average effect of the intervention across facilities while preserving the true timing of implementation at each site. The median number of preintervention and postintervention months per facility was 6 and 9, respectively.

Effect sizes are reported as incidence rate ratios and in clinically interpretable estimated per cent change for both the immediate level change and post-intervention trend change.

Most outcomes were counts and were modelled using negative binomial regression to account for overdispersion. Facility fixed effects were included to control for time-invariant differences between facilities (eg, baseline volume or catchment characteristics). For malaria positivity, an offset for the number of persons with fever was included to model the rate appropriately.

Because observations were repeated monthly within facilities, cluster-robust standard errors at the facility level were used to account for correlation of observations within facilities over time. Cluster-robust standard errors were chosen rather than specifying a parametric autocorrelation structure (eg, AR(1)) to avoid imposing assumptions about the form of serial correlation in a relatively short time series.

Seasonal terms were not included. The analytic period consisted of 15 months, which does not provide sufficient repeated annual cycles to reliably estimate seasonal effects independent of intervention-related changes. Given the short time series and staggered implementation, inclusion of seasonal indicators risked overparameterisation and was unlikely to improve model validity.

We assessed whether concurrent state-wide programmes or policy changes occurred during the study period. No major state-wide maternal health initiatives were identified that aligned with the intervention timeline or plausibly explained level or slope changes. However, national adoption of WHO-recommended ANC guidelines occurred prior to implementation and likely influenced service use trends through ANC-focused institutional directives and mass media promotion.

### Methodological limitations

The use of aggregate facility-level data and the observational study design without a control group present methodological limitations in this study. For instance, routine DHIS2 data, despite significant project efforts to improve data quality, may still have challenges such as with potential misclassification and underreporting. In addition, limited preintervention time series and absence of a formal control group to fully separate project effects from national trends require the results to be interpreted with caution. Furthermore, lack of patient-reported satisfaction and experience data and the inability to isolate effects among G-ANC participants versus non-participants narrow the scope of the analysis.

## Results

### Implementation outputs

Ninety-one health facilities started implementing G-ANC in the first 2 months of the project and 137 in the first three. During the period of implementation, 33 883 pregnant women attended a first ANC visit of which 17 706 (52%) were enrolled in G-ANC. Only in the final months did the percentage of women receiving G-ANC exceed 50% of all women attending ANC. Conventional ANC was ongoing through implementation. By the end of the project, a total of 17 706 women received G-ANC. The number of G-ANC meetings increased steadily from only 200 in the first month to over 1000 by the final month.

Out of the 301 HCWs trained to lead the G-ANC, 249 were community health workers; only 31 were nurses or midwives. Of the 407 government community outreach workers who were trained to increase awareness and refer pregnant women to the new G-ANC, only 72% ever referred a pregnant woman to G-ANC and by the end of the project only 16% were active. Overall, the outreach workers only contributed 6% of the total G-ANC attendance. Low and irregular payments to outreach workers interfered with their optimal participation.

### Project effects on maternal health service delivery measures

As the interrupted time series analysis model included time, level and slope to account for the staggered roll out across health facilities, the level and slope change variables were aligned with each facility’s specific implementation date. This approach allowed for estimation of the average intervention effect across sites while maintaining accuracy regarding the timing of implementation at each facility.

Results indicated significant changes in four outcomes:

- ANC first visit
  - *Increased in level* after the intervention.
  - *Decreased in slope* after the intervention.
- ANC fourth visit
  - *Increased in level* after the intervention.
- Facility births
  - *Increased in level* after the intervention.
  - *Decreased in slope* after the intervention.
- Live births
  - *Increased in level* after the intervention.
  - *Decreased in slope* after the intervention.

**Figure 1:**
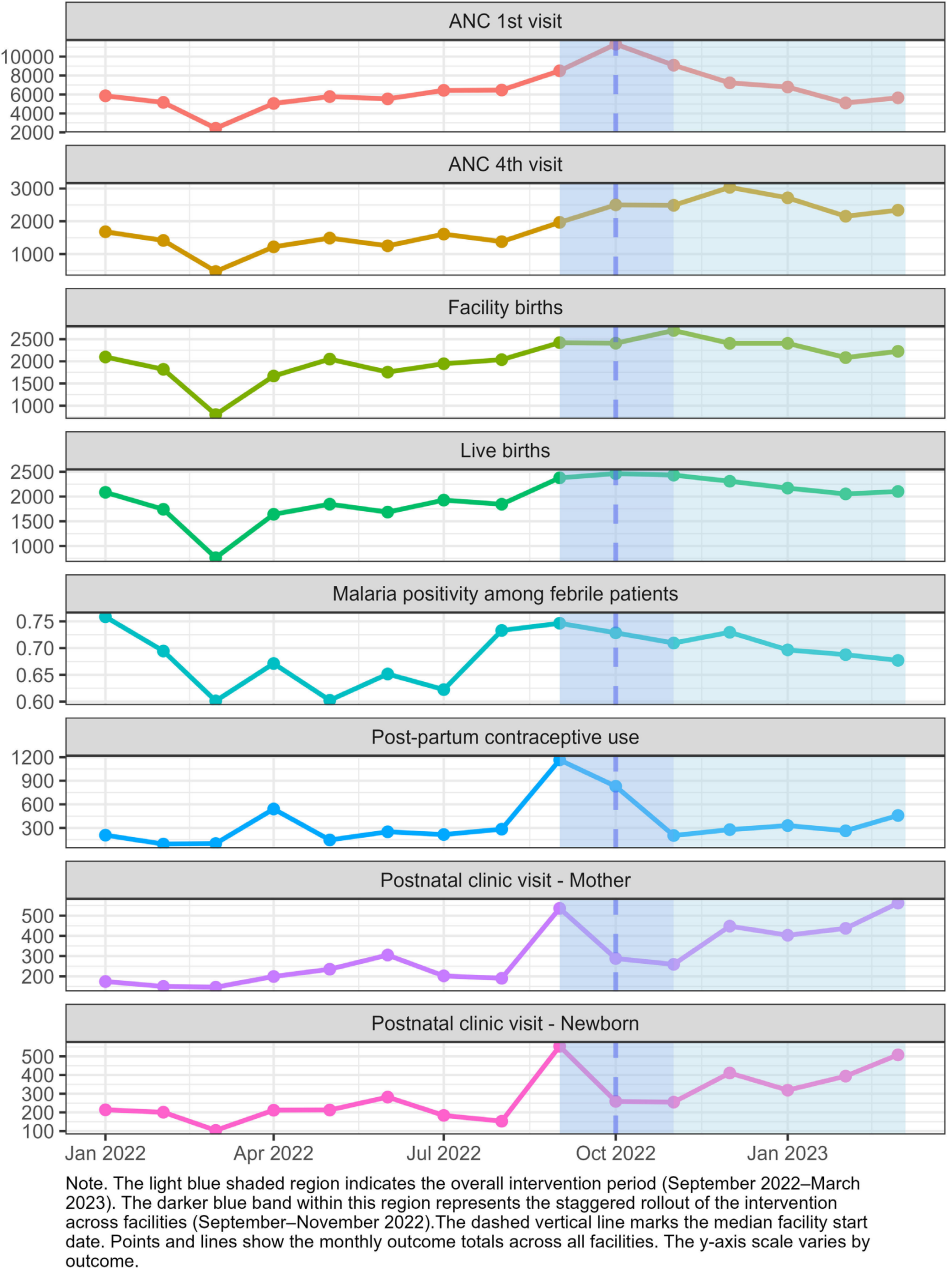
Trends in each of the eight outcome variables over time, with a shaded region indicating the staggered start of the intervention across participating facilities (September–November 2022) (placeholder for figure 1). ANC, antenatal care.

The interrupted time series model identified significant changes in four individual indicators: ANC first visit, ANC fourth visit, facility births and live births (table 1). For ANC first visit, there was a 37% immediate increase when the intervention initiated and a subsequent 16% decline per month over the following months of the intervention. ANC fourth visits also showed a significant 56% immediate increase when G-ANC was initiated and the trend in numbers of visits stayed at this level for the rest of the intervention, showing durable change. Facility births increased 21% at the beginning of the intervention and declined by 7% per month after that. Similarly, live births increased by 15% at the time G-ANC was initiated and then declined 6% per month after that. Both facility births and live births experienced significant level increases after the intervention (p=0.028 and p=0.033, respectively). Despite these significant effects following the initiation of the intervention, the trends show a distinct levelling off and decline across all significant measures except ANC fourth visit during the implementation period. In addition, the outcome measures of malaria positivity, postpartum contraceptive use and maternal and newborn postnatal clinical visits—did not show statistically significant changes during the study period.

**Table 1:** Interrupted time series model results

Outcome	Term	Estimate	SE	P value	IRR	Effect
ANC first visit	Time	0.08	0.01	<0.001	1.08	
	Level	0.31	0.07	<0.001	1.37	Immediate change: +37%
	Slope	−0.17	0.02	<0.001	0.84	Change in trend: −16% per month
ANC fourth visit	Time	0.05	0.02	0.005	1.05	
	Level	0.44	0.14	0.001	1.56	Immediate change: +56%
	Slope	−0.03	0.03	0.381	0.97	
Facility births	Time	0.05	0.01	<0.001	1.05	
	Level	0.19	0.06	0.002	1.21	Immediate change: +21%
	Slope	−0.07	0.02	<0.001	0.93	Change in trend: −7% per month
Live births	Time	0.05	0.01	<0.001	1.05	
	Level	0.14	0.05	0.008	1.15	Immediate change: +15%
	Slope	−0.06	0.02	<0.001	0.94	Change in trend: −6% per month
Malaria positivity	Time	0.01	0.02	0.739	1.01	
	Level	0.12	0.08	0.123	1.12	
	Slope	−0.05	0.03	0.061	0.95	
Postpartum contraceptive use	Time	0.14	0.11	0.196	1.15	
	Level	−0.22	0.84	0.798	0.81	
	Slope	−0.12	0.19	0.539	0.89	
Postnatal clinical visit—mother	Time	0.09	0.03	0.001	1.10	
	Level	−0.04	0.20	0.840	0.96	
	Slope	0.08	0.06	0.167	1.09	
Postnatal clinical visit—newborn	Time	0.09	0.04	0.017	1.09	
	Level	−0.14	0.26	0.577	0.87	
	Slope	0.10	0.07	0.191	1.10	

ANC, antenatal care; IRR, incidence rate ratios.

## Discussion

The aim of this study was to measure the effects of the large-scale, short-term G-ANC project on antenatal contacts, facility births, live births, malaria positivity, postpartum contraception and PNC. Results showed that first and fourth ANC contacts significantly increased following the introduction of G-ANC, as did facility births and live births. Malaria positivity declined but the change was not significant. Project effects on postpartum contraception and PNC were also not significant.

Increasing live birth is a significant finding particularly for an ANC project that did not include an intrapartum component. Other literature has found that increasing the quality of ANC does have an impact on stillbirths. In addition, increases in facility birth are often associated with better outcomes.^16^ It is notable that these improvements were observed in this rural setting, where only community health workers were used.

Considerable thought has gone into defining SBAs which include those providing ANC. The International Confederation of Midwives has a standard curriculum and competencies.^17^ To respond to gaps in SBAs, Nigeria has a national task shifting policy that enables community health workers to provide maternity care notably in rural areas.^18^ This project used the existing government community health workers in the role of midwives with overall improved outcomes. The literature finds mixed results for community health workers in the role of midwives.^19^,^21^ Community health workers have been shown to increase facility birth rates but birth outcomes are a concern. Although CHWs gaps in expertise may explain why this project did not change family planning and malaria outcomes, community health workers were able to be supported to implement this ANC project which is associated with improvement in health outcomes, in particular durable improvement for ANC fourth visit. Research is needed to determine the best use of community health workers, particularly in the lowest resourced settings.

The broad stakeholder engagement that brought resources, such as essential supplies to facilities and health insurance to pregnant women, is thought to have strengthened implementation. The impact was likely in part a result of leveraging all stakeholders and related programmes and building a strong network.^22^,^24^ The synergies established is an aspect of project implementation that is often overlooked.^25^ Challenges that interfere with synergising include fragmentation within weak health systems and competition between partners. Although all these concerns existed in this context, the project maximised the benefits of collaboration and benefitted from partner activities.

Intensive provider training, support for mentoring and supervision and strengthening of data collection and use were also likely to have contributed to the project’s results, despite the only 6 month implementation period. The significant immediate improvements in certain measures are especially notable given the baseline finding that critical gaps in life-saving services, including essential equipment to prevent maternal death and 24/7 care availability, were ubiquitous. Research exposes similar findings in many low-resource settings. Using demographic and health surveys from 10 low-income and middle-income countries, Benova *et al* found that in the poorest settings, only 10%–15% of health facilities provide the basic components of ANC.^26^ However, in the Benova *et al* article, Nigeria ranked higher than other countries with rates of functional ANC as high as 50%. Globally, blood pressure monitoring is the most common component of ANC. In our baseline study, availability of blood pressure equipment had significant gaps. At the same time, the levelling off that we see could be attributed to a dropping off of enthusiasm, notably with the community health workers who were raising awareness, as well as full engagement of involved populations. Limitations to the project that also could have contributed to the weaker results in some measures included local insecurity, inadequate skilled health workers, significant supply gaps, ongoing fees for services and abrupt closure due to the stoppage of funding.

One aspect of the programme that likely had a positive contribution and also could have been stronger is mentorship. Mentorship as a way to ensure implementation of training is supported in the literature.^22^ Research in low-income settings shows that mentorship can facilitate behaviour change among HCWs and managers. Mentors are acknowledged in the literature as effective for improving quality implementation and facilitating improvements in clinical care in low-income and middle-income countries.^23 24^ Using existing government staff in the role of mentors has a precedent in literature and has been found to contribute to quality programming. Although there are advantages to using mentors external to the system, who are not burdened by other duties and may be less influenced by political influences, using those within the system is more likely to support sustainability. The literature finds that mentoring can comprise both clinical and facility-wide interventions aimed to capacitate and create enabling environments for quality care.^23^ Mentoring enlists advocacy, modelling and problem solving to achieve quality improvements.

That this project focused on both direct service delivery and broader system strengthening is an important aspect to recognise. The project worked on strengthening the national supply chain and directly provided blood pressure cuffs to facilities. Projects often struggle with the dilemma, highlighted in the health system strengthening literature, of donating commodities separately from government’s supply chain, as donations in general are not sustainable and may even demotivate system strengthening.^27^ This is relevant to point out because providing supplies was important for implementation—quality ANC could not have taken place otherwise. Ensuring supplies while also strengthening systems was critical and is a common dilemma for projects.

The strong emphasis on data collection, monitoring and evaluation was another important aspect of the project. The project leveraged existing government employees to strengthen data management through a DHIS2 tracker who were active, collaborative partners. All pregnant women were able to be followed from registration through the post-partum period. While project monitoring is essential to understanding its impact, it is often limited by weak host country data collection systems. This project stressed the importance of continuous monitoring to ensure quality and worked closely within the government systems to ensure sustainability. This focus is recognised in the literature as a component of ensuring quality implementation.^28^

This project had an unanticipated finding with regard to scheduling tools. Project facilities did not have scheduling systems which presented challenges for arranging G-ANC sessions. The need to introduce a scheduling tool to improve quality or expand on ANC is found in the literature.^29^ Although scheduling does facilitate more efficient care, particularly for the beneficiary, functionalising scheduling tools in low-resource settings has complexities including that many women coming for care may not have smart phones so the system needs to be hybrid, and also that if the larger health system is not using a scheduling system, the providers themselves are burdened with negotiating the logistics.

While the rigorous analysis indicates an association between the project intervention and maternal health measures, other influences cannot be ruled out. For instance, increasing trends in the measures with significant change in this analysis were noted prior to the project’s initiation. A contributing factor to this may have been national-level adoption of WHO-recommended ANC guidelines prior to state-level project implementation. At the same time, the project had much more intensive, local-level operations than the national ANC efforts did, and the local-level support likely played a stronger role.

Overall, this project highlights the significant gaps in primary health care availability, quality and service utilisation that are common in low-resource settings and sheds light on effective quality improvements. Despite challenges, this relatively brief G-ANC project made a difference.

## Conclusions

In spite of numerous limitations, the introduction of a system strengthening G-ANC project in Niger State, alongside broad stakeholder partnerships and strong facility mentoring, was associated with increased ANC visits, facility births and live births. Given recent national-level adoption of ANC guidelines, other factors may also have contributed. Strengthening the health system in the areas of data, service delivery, supplies and equipment and social insurance made it possible to successfully implement a G-ANC service delivery model. Further work is needed to increase availability of skilled providers, expand health insurance, secure essential supplies, pay health workers in a timely manner and institute facility-based appointment scheduling systems.

## Supplementary material

10.1136/bmjgh-2025-022038 online supplemental file 1

## Data Availability

Data are available upon reasonable request.
