# Supplementary material for: The introduction of group antenatal care in northern Nigeria: an implementation research study analysing changes in service use
Source: BMJ Glob Health. 2026 Jul 14;11(7):e022038. doi: 10.1136/bmjgh-2025-022038 (PMC13374479; doi:10.1136/bmjgh-2025-022038)
Supplement: online supplemental file 1 [file bmjgh-11-7-s001.docx]

### Instructions for Authors Text (under [Original research](https://gh.bmj.com/pages/authors#original_research)):

Authors submitting research from international partnerships between [high-income countries and low- and/or middle-income countries](https://datahelpdesk.worldbank.org/knowledgebase/articles/906519-world-bank-country-and-lending-groups) are required to submit an author reflexivity statement, in line with our goals to [improve equitable inclusion in global health research](https://gh.bmj.com/content/6/10/e007632). Please complete the Author Reflexivity Statement using the provided Word or PDF format. Once finished, upload it as the ‘Supplemental Material’ file for publication.

### Instructions for EPA (to be added to EPA checklist for original submissions)

- Check the author list of ‘Original research’ article types to identify whether there are authors from both [high-income countries and low- and/or middle-income countries](https://datahelpdesk.worldbank.org/knowledgebase/articles/906519-world-bank-country-and-lending-groups).
- If yes, check that a reflexivity statement has been included as ‘Supplemental Material’ file type
- If included as ‘Supplemental Material for Editors only’, change to ‘Supplemental Material’and inform authors
- If no reflexivity statement, return manuscript to authors with the full text from the Instructions for Authors.

### Questions for Reviewers and Editors - to be emailed to Associate Editors? Included on Reviewer form/invite?

- Has the research team engaged constructively with the reflexivity statement?
- Have the research partners co-developed the research study?
- Does the study address priority research questions for the LMIC partner(s)?
- Is there a LMIC partner who is the first or last author? If not, what is the explanation?
- How have LMIC early career researchers been incorporated as authors?
- How are data shared with LMIC partners to address research needs?
- Is there open access funding to improve publication dissemination?

### BMJ Global Health Author Reflexivity Statement

Adapted from Morton, B., Vercueil, A., Masekela, R., Heinz, E., Reimer, L., Saleh, S., Kalinga, C., Seekles, M., Biccard, B., Chakaya, J., Abimbola, S., Obasi, A. and Oriyo, N. (2022), Consensus statement on measures to promote equitable authorship in the publication of research from international partnerships. Anaesthesia, 77: 264-276. <https://doi.org/10.1111/anae.15597>

| **Study conceptualisation** | |
| --- | --- |
| 1. How does this study address local research and policy priorities? | Maternal morbidity and mortality are leading public health challenges in Nigeria and ensuring more pregnanct women receive antenatal care is a national health priority. |
| 1. How were local researchers involved in study design? | Local researchers designed the data collection questions and tools, collected the data, and did the initial analysis. |
| **Research management** | |
| 1. How has funding been used to support the local research team(s)? | The local members of the researcher team were long-term local staff of Project Hope who oversaw project design and implementation. The paper’s lead author is a local researcher who was hired to lead the data collection and analysis for the project team. |
| **Data acquisition and analysis** | |
| 1. How are research staff who conducted data collection acknowledged? | The staff who collected the data are acknowledged in the Data Sources and Collection section in the line “Data collection and analysis were conducted by the project monitoring and evaluation team.” |
| 1. How have members of the research partnership been provided with access to study data? | All co-authors meet ICMJE authorship criteria, which includes reviewing the study data and taking responsibility for it. The statistical analysis in the paper has been shared among all authors who have disseminated to other members of the local project team. |
| 1. How were data used to develop analytical skills within the partnership? | The lead author who developed the initial report with descriptive statistics provided input on the design for the inferential analysis. They were also invited to lead the statistical analysis and in the end decided to play a supportive role. |
| **Data interpretation** | |
| 1. How have research partners collaborated in interpreting study data? | All study co-authors reviewed initial and final statistical analyses and had opportunities to provide comments during their reviews. |
| **Drafting and revising for intellectual content** | |
| 1. How were research partners supported to develop writing skills? | Research partners were invited to write parts of the manuscript with revision and editing support by more experienced writers and editors. |
| 1. How will research products be shared to address local needs? | Draft, preprint, and final versions of the manuscript will be shared with all local members of the Project HOPE team in Nigeria who have internal project management systems for dissemination and learning. |
| **Authorship** | |
| 1. How is the leadership, contribution and ownership of this work by LMIC researchers recognised within the authorship? | The lead author role is held by a local member of the research team who led the initial data collection and analysis. All other key members of the local research team who met the criteria for authorship were included as coauthors. |
| 1. How have early career researchers across the partnership been included within the authorship team? | Career stage was not an explicit criteria applied to the selection of authors. |
| 1. How has gender balance been addressed within the authorship? | The majority of the authors are women and among local members of the research team the is an equal female/male gender balance. |
| **Training** | |
| 1. How has the project contributed to training of LMIC researchers? | This project substantially contributed to training LMIC researchers as these individuals made up the entirety of the local research team that designed and conducted the baseline and endline analyses and this work was supported by experienced international research and evaluation staff with Project Hope. |
| **Infrastructure** | |
| 1. How has the project contributed to improvements in local infrastructure? | Local infrastructure development was not part of this project apart from the provision of certain essential antenatal care supplies such as blood pressure cuffs |
| **Governance** | |
| 1. What safeguarding procedures were used to protect local study participants and researchers? | Project Hope’s human resource policies, which apply best practices for staff safety and labor standards, were followed for all staff and consultants at all stages of this project. |
